# Author Correction: Polyelectrolyte interactions enable rapid association and dissociation in high-affinity disordered protein complexes

**DOI:** 10.1038/s41467-023-41573-3

**Published:** 2023-09-21

**Authors:** Andrea Sottini, Alessandro Borgia, Madeleine B. Borgia, Katrine Bugge, Daniel Nettels, Aritra Chowdhury, Pétur O. Heidarsson, Franziska Zosel, Robert B. Best, Birthe B. Kragelund, Benjamin Schuler

**Affiliations:** 1 Department of Biochemistry, University of Zurich, Zurich, Switzerland; 2 Structural Biology and NMR Laboratory (SBiNLab) and REPIN, Department of Biology, Ole Maaloes Vej 5, University of Copenhagen, 2200 Copenhagen, Denmark; 3 Laboratory of Chemical Physics, National Institute of Diabetes and Digestive and Kidney Diseases, National Institutes of Health, Bethesda, MD 20892-0520 USA; 4 Department of Physics, University of Zurich, Zurich, Switzerland; 5 Present Address: Department of Structural Biology, St. Jude Children’s Research Hospital, Memphis, TN 38105 USA; 6 Present Address: Department of Biochemistry, Science Institute, University of Iceland, Dunhagi 3, 107 Reykjavík, Iceland; 7 Present Address: Novo Nordisk A/S, Novo Nordisk Park, 2760 Måløv, Denmark

**Keywords:** Kinetics, Single-molecule biophysics, Computational models

Correction to: *Nature Communications* 10.1038/s41467-020-18859-x, published online 12 November 2020

The original Article contained errors in text in the Methods section, and in Equations (2), (4), (6), (11), and (15). All calculations were performed with the correct equations, so none of the reported results or conclusions are affected.

In the section “Single-molecule fluorescence spectroscopy” of the Methods section, the second sentence incorrectly read ‘After passing through a 100-mm pinhole, …’. The correct version states ‘100-μm pinhole’ instead of ‘100-mm pinhole’.

Equation (2) incorrectly read

$$\theta ({c}_{{{{\rm{H}}}}}^{{{{\rm{tot}}}}})=\frac{({c}_{{{{\rm{H}}}}}^{{{{\rm{tot}}}}}+{K}_{{{{\rm{D}}}}}^{\ast }+{c}_{{{{\rm{P}}}}\ast }^{{{{\rm{tot}}}}})+\sqrt{{({c}_{{{{\rm{H}}}}}^{{{{\rm{tot}}}}}+{K}_{{{{\rm{D}}}}}^{\ast }+{c}_{{{{\rm{P}}}}\ast }^{{{{\rm{tot}}}}})}^{2}-4{c}_{{{{\rm{H}}}}}^{{{{\rm{tot}}}}}{c}_{{{{\rm{P}}}}\ast }^{{{{\rm{tot}}}}}}}{2{c}_{{{{\rm{P}}}}\ast }^{{{{\rm{tot}}}}}}$$

The correct form of Equation (2) is

$$\theta ({c}_{{{{\rm{H}}}}}^{{{{\rm{tot}}}}})=\frac{({c}_{{{{\rm{H}}}}}^{{{{\rm{tot}}}}}+{K}_{{{{\rm{D}}}}}^{\ast }+{c}_{{{{\rm{P}}}}\ast }^{{{{\rm{tot}}}}})-\sqrt{{({c}_{{{{\rm{H}}}}}^{{{{\rm{tot}}}}}+{K}_{{{{\rm{D}}}}}^{\ast }+{c}_{{{{\rm{P}}}}\ast }^{{{{\rm{tot}}}}})}^{2}-4{c}_{{{{\rm{H}}}}}^{{{{\rm{tot}}}}}{c}_{{{{\rm{P}}}}\ast }^{{{{\rm{tot}}}}}}}{2{c}_{{{{\rm{P}}}}\ast }^{{{{\rm{tot}}}}}}$$

Equation (4) incorrectly read

$${\theta }_{{{{\rm{coupled}}}}}({c}_{{{{\rm{P}}}}}^{{{{\rm{tot}}}}})=\frac{{c}_{{{{\rm{H}}}}}^{{{{\rm{tot}}}}}{K}_{{{{\rm{D}}}}}^{\ast }+{c}_{{{{\rm{P}}}}}^{{{{\rm{tot}}}}}{K}_{{{{\rm{D}}}}}^{\ast }-2{c}_{{{{\rm{H}}}}}^{{{{\rm{tot}}}}}{K}_{{{{\rm{D}}}}}-{K}_{{{{\rm{D}}}}}^{\ast }{K}_{{{{\rm{D}}}}}+{K}_{{{{\rm{D}}}}}^{\ast }\sqrt{{(-{c}_{{{{\rm{H}}}}}^{{{{\rm{tot}}}}}+{K}_{{{{\rm{D}}}}}+{c}_{{{{\rm{P}}}}}^{{{{\rm{tot}}}}})}^{2}+4{c}_{{{{\rm{H}}}}}^{{{{\rm{tot}}}}}{K}_{{{{\rm{D}}}}}}}{2[{c}_{{{{\rm{H}}}}}^{{{{\rm{tot}}}}}({K}_{{{{\rm{D}}}}}^{\ast }-{K}_{{{{\rm{D}}}}})+{K}_{{{{\rm{D}}}}}^{\ast }(-{c}_{{{{\rm{P}}}}}^{{{{\rm{tot}}}}}-{K}_{{{{\rm{D}}}}}+{K}_{{{{\rm{D}}}}}^{\ast })]}$$

The correct form of Equation (4) is

$${\theta }_{{{{\rm{coupled}}}}}({c}_{{{{\rm{P}}}}}^{{{{\rm{tot}}}}})=\frac{{c}_{{{{\rm{H}}}}}^{{{{\rm{tot}}}}}{K}_{{{{\rm{D}}}}}^{\ast }-{c}_{{{{\rm{P}}}}}^{{{{\rm{tot}}}}}{K}_{{{{\rm{D}}}}}^{\ast }-2{c}_{{{{\rm{H}}}}}^{{{{\rm{tot}}}}}{K}_{{{{\rm{D}}}}}-{K}_{{{{\rm{D}}}}}^{\ast }{K}_{{{{\rm{D}}}}}+{K}_{{{{\rm{D}}}}}^{\ast }\sqrt{{(-{c}_{{{{\rm{H}}}}}^{{{{\rm{tot}}}}}+{K}_{{{{\rm{D}}}}}+{c}_{{{{\rm{P}}}}}^{{{{\rm{tot}}}}})}^{2}+4{c}_{{{{\rm{H}}}}}^{{{{\rm{tot}}}}}{K}_{{{{\rm{D}}}}}}}{2[{c}_{{{{\rm{H}}}}}^{{{{\rm{tot}}}}}({K}_{{{{\rm{D}}}}}^{\ast }-{K}_{{{{\rm{D}}}}})+{K}_{{{{\rm{D}}}}}^{\ast }(-{c}_{{{{\rm{P}}}}}^{{{{\rm{tot}}}}}-{K}_{{{{\rm{D}}}}}+{K}_{{{{\rm{D}}}}}^{\ast })]}$$

Equation (6) incorrectly read

$$\theta ({c}_{{{{\rm{H}}}}}^{{{{\rm{tot}}}}})=\frac{({c}_{{{{\rm{H}}}}}^{{{{\rm{tot}}}}}+{K}_{{{{\rm{D}}}}}^{{{{\rm{PHH}}}}}+{c}_{{{{{\rm{P}}}}}^{\ast }}^{{{{\rm{tot}}}}})+\sqrt{{({c}_{{{{\rm{H}}}}}^{{{{\rm{tot}}}}}+{K}_{{{{\rm{D}}}}}^{{{{\rm{PHH}}}}}+{c}_{{{{{\rm{P}}}}}^{\ast }}^{{{{\rm{tot}}}}})}^{2}-4{c}_{{{{\rm{H}}}}}^{{{{\rm{tot}}}}}{c}_{{{{{\rm{P}}}}}^{\ast }}^{{{{\rm{tot}}}}}}}{2{c}_{{{{{\rm{P}}}}}^{\ast }}^{{{{\rm{tot}}}}}}$$

The correct form of Equation (6) is

$$\theta ({c}_{{{{\rm{H}}}}}^{{{{\rm{tot}}}}})=\frac{({c}_{{{{\rm{H}}}}}^{{{{\rm{tot}}}}}+{K}_{{{{\rm{D}}}}}^{{{{\rm{PHH}}}}}+{c}_{{{{{\rm{P}}}}}^{\ast }}^{{{{\rm{tot}}}}})-\sqrt{{({c}_{{{{\rm{H}}}}}^{{{{\rm{tot}}}}}+{K}_{{{{\rm{D}}}}}^{{{{\rm{PHH}}}}}+{c}_{{{{{\rm{P}}}}}^{\ast }}^{{{{\rm{tot}}}}})}^{2}-4{c}_{{{{\rm{H}}}}}^{{{{\rm{tot}}}}}{c}_{{{{{\rm{P}}}}}^{\ast }}^{{{{\rm{tot}}}}}}}{2{c}_{{{{{\rm{P}}}}}^{\ast }}^{{{{\rm{tot}}}}}}$$

Equation (11) incorrectly read

$$\langle E\rangle (t)=(1-{p}_{{{{\rm{same}}}}}(t)){\langle E\rangle }_{{{{\rm{eq}}}}}+{p}_{{{{\rm{same}}}}}(t)({\langle E\rangle }_{{{{\rm{eq}}}}}-\langle E\rangle (0)){e}^{-{k}_{{{{\rm{ex}}}}}t}$$

The correct form of Equation (11) is

$$\langle E\rangle (t)=(1-{p}_{{{{\rm{same}}}}}(t)){\langle E\rangle }_{{{{\rm{eq}}}}}+{p}_{{{{\rm{same}}}}}(t)[{\langle E\rangle }_{{{{\rm{eq}}}}}+(\langle E\rangle (0)-{\langle E\rangle }_{{{{\rm{eq}}}}}){e}^{-{k}_{{{{\rm{ex}}}}}t}]$$

Equation (15) incorrectly read

$${\tau }_{{{{\rm{high}}}}}^{{{{\rm{P}}}}}=\frac{2{c}_{{{{\rm{H}}}}}({k}_{{{{\rm{off}}}}}^{{{{\rm{PPH}}}}}+{c}_{{{{\rm{P}}}}}{k}_{{{{\rm{on}}}}}^{{{{\rm{PPH}}}}})+{c}_{{{{\rm{PH}}}}}{k}_{{{{\rm{on}}}}}^{{{{\rm{PPH}}}}}(2{k}_{{{{\rm{off}}}}}+{k}_{{{{\rm{off}}}}}^{{{{\rm{PPH}}}}}+2{c}_{{{{\rm{P}}}}}{k}_{{{{\rm{on}}}}}^{{{{\rm{PPH}}}}})}{{k}_{{{{\rm{off}}}}}^{{{{\rm{PPH}}}}}(2{k}_{{{{\rm{off}}}}}+{c}_{{{{\rm{P}}}}}{k}_{{{{\rm{on}}}}}^{{{{\rm{PPH}}}}})({c}_{{{{\rm{H}}}}}{k}_{{{{\rm{on}}}}}+{c}_{{{{\rm{PH}}}}}{k}_{{{{\rm{on}}}}}^{{{{\rm{PPH}}}}})}$$

The correct form of Equation (15) is

$${\tau }_{{{{\rm{high}}}}}^{{{{\rm{P}}}}}=\frac{2{c}_{{{{\rm{H}}}}}{k}_{{{{\rm{on}}}}}({k}_{{{{\rm{off}}}}}^{{{{\rm{PPH}}}}}+{c}_{{{{\rm{P}}}}}{k}_{{{{\rm{on}}}}}^{{{{\rm{PPH}}}}})+{c}_{{{{\rm{PH}}}}}{k}_{{{{\rm{on}}}}}^{{{{\rm{PPH}}}}}(2{k}_{{{{\rm{off}}}}}+{k}_{{{{\rm{off}}}}}^{{{{\rm{PPH}}}}}+2{c}_{{{{\rm{P}}}}}{k}_{{{{\rm{on}}}}}^{{{{\rm{PPH}}}}})}{{k}_{{{{\rm{off}}}}}^{{{{\rm{PPH}}}}}(2{k}_{{{{\rm{off}}}}}+{c}_{{{{\rm{P}}}}}{k}_{{{{\rm{on}}}}}^{{{{\rm{PPH}}}}})({c}_{{{{\rm{H}}}}}{k}_{{{{\rm{on}}}}}+{c}_{{{{\rm{PH}}}}}{k}_{{{{\rm{on}}}}}^{{{{\rm{PPH}}}}})}$$

This has been corrected in both the PDF and HTML versions of the Article.

