# Supplementary material for: Polyelectrolyte interactions enable rapid association and dissociation in high-affinity disordered protein complexes
Source: Nat Commun. 2020 Nov 12;11:5736. doi: 10.1038/s41467-020-18859-x (PMC7661507; doi:10.1038/s41467-020-18859-x)
Supplement: Supplementary file 1 — Supplementary Information [file 41467_2020_18859_MOESM1_ESM.pdf]

Supplementary Information for

Polyelectrolyte interactions enable rapid association and dissociation in high-affinity disordered protein complexes

Andrea Sottini[a], Alessandro Borgia[a,1], Madeleine B. Borgia[a,1], Katrine Bugge[b], Daniel Nettels[a], Aritra Chowdhury[a], Pétur Heidarsson[a,2], Franziska Zosel[a,3] , Robert B. Best[c]*, Birthe B. Kragelund[b]*, Benjamin Schuler[a,d]*

[a]Department of Biochemistry, University of Zurich, Zurich, Switzerland

[b]Structural Biology and NMR Laboratory (SBiNLab) and REPIN, Department of Biology, Ole Maaloes Vej 5, University of Copenhagen, 2200 Copenhagen, Denmark;

[c]Laboratory of Chemical Physics, National Institute of Diabetes and Digestive and Kidney Diseases, National Institutes of Health, Bethesda, Maryland 20892-0520, USA

[d]Department of Physics, University of Zurich, Zurich, Switzerland

* To whom correspondence should be addressed

Email: bbk@bio.ku.dk, robert.best2@nih.gov, schuler@bioc.uzh.ch

---

[1] Current Address: Department of Structural Biology, St. Jude Children's Research Hospital, Memphis, TN 38105, USA
[2] Current Address: Department of Biochemistry, Science Institute, University of Iceland, Dunhagi 3, 107 Reykjavík, Iceland
[3] Current Address: Novo Nordisk A/S, Novo Nordisk Park, 2760 Måløv, Denmark

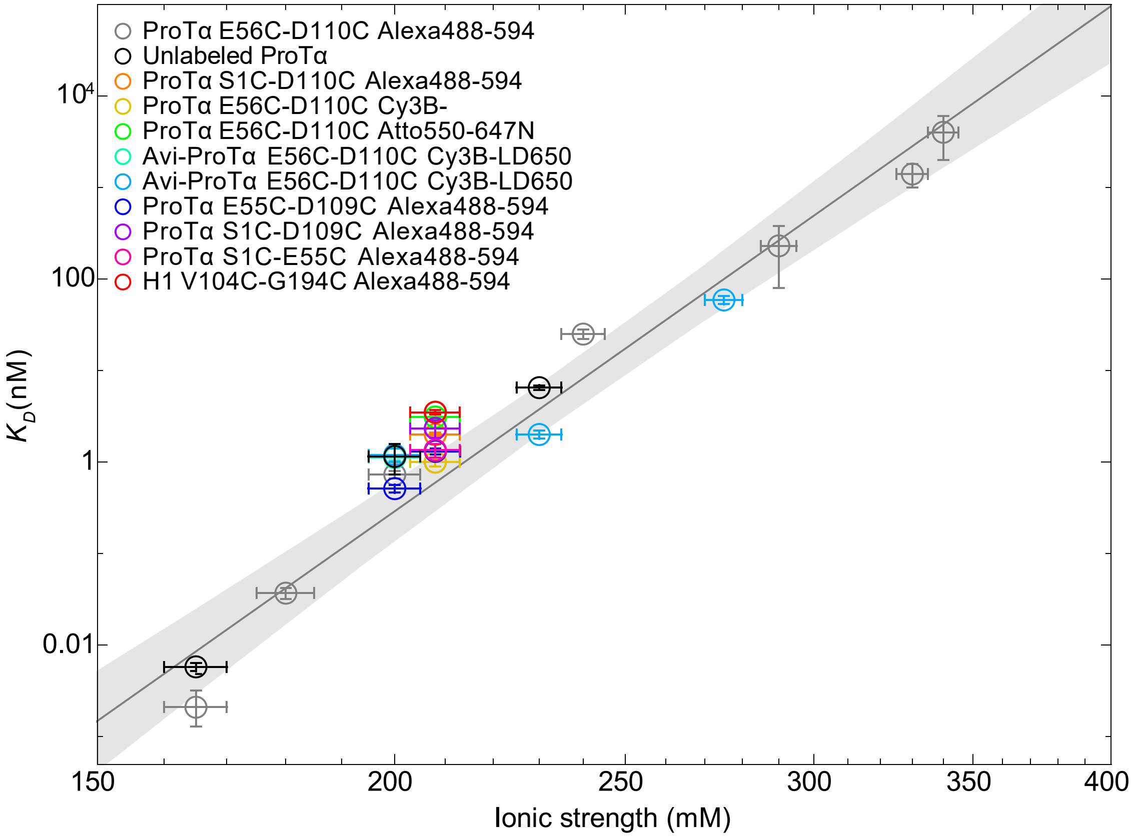

Supplementary Figure 1. ProTα-H1 affinity is robust to fluorophore labeling, surface immobilization, and slight sequence variations, but highly dependent on ionic strength. Measured equilibrium dissociation constants (K_D) for different ProTα and H1 variants as a function of ionic strength (see legend and Supplementary Table 1). A fit to the Lohman-Record model[1,2] was used to estimate the number of counter ions released upon binding (18 ± 1, solid line; shaded band indicates 67 % confidence interval). K_D values for surface-immobilized ProTα were measured with Avi-tagged ProTα (Figure 3). K_D values for fluorophore-labeled ProTα were measured with unlabeled H1, values for fluorophore-labeled H1 with unlabeled ProTα. The value denoted "Unlabeled ProTα" is from competition titrations (Figure 1c). Horizontal error bars indicate our estimate of the uncertainty in ionic strength while vertical error bars are the standard error of the fits reported in Supplementary Table 1.

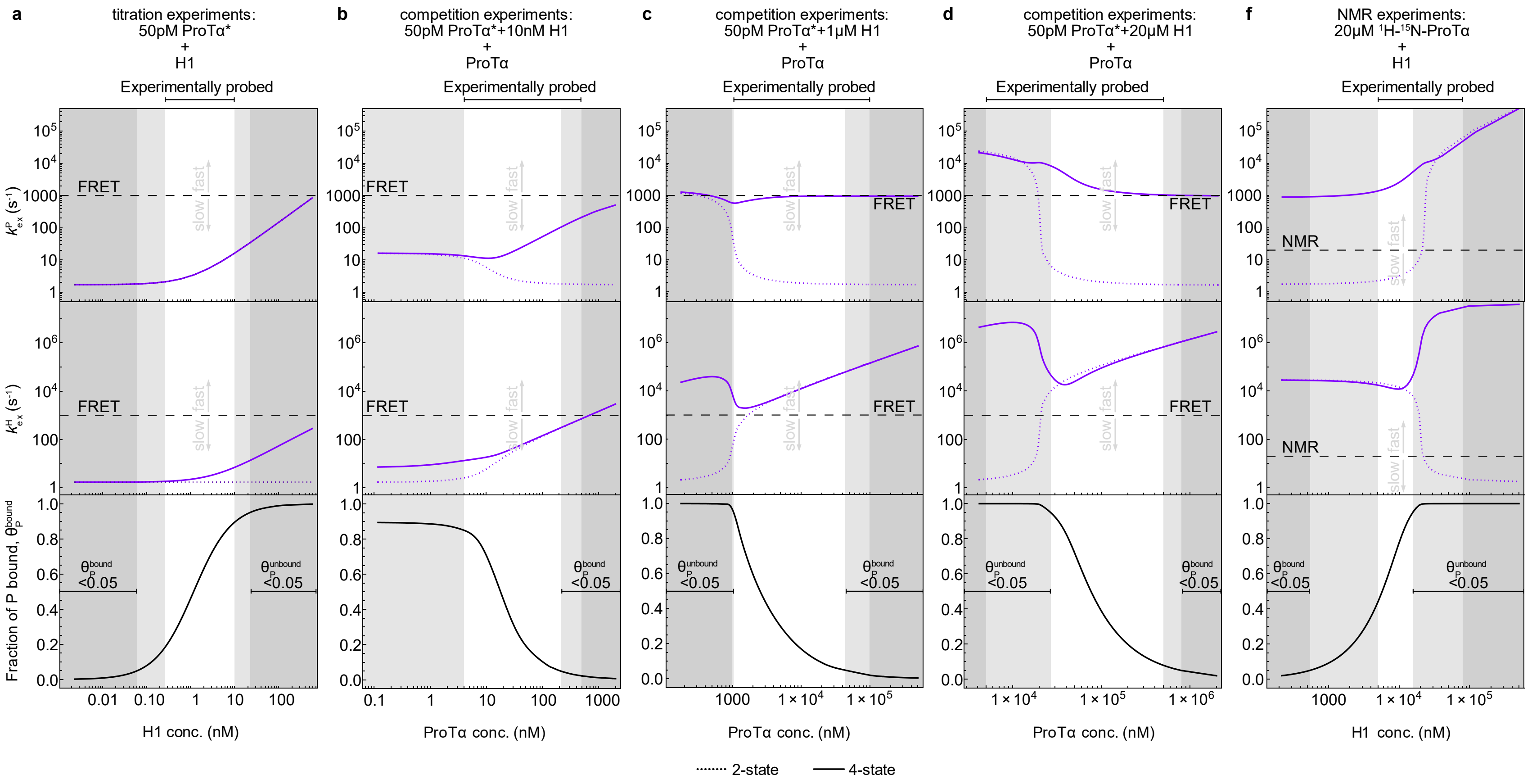

$$P+H \underset{k_{off}^{PH}=1.7\ s^{-1}}{\overset{k_{on}^{PH}=1.45\times10^{9}\ M^{-1}\ s^{-1}}{\rightleftharpoons}} PH \qquad K_D=1.2nM$$

$$PH+P \underset{k_{off}^{PPH}=1.9\times10^{3}\ s^{-1}}{\overset{k_{on}^{PPH}=0.53\times10^{9}\ M^{-1}\ s^{-1}}{\rightleftharpoons}} PPH \qquad K_D=3.5\mu M$$

$$PH+H \underset{k_{off}^{PHH}=6.4\times10^{3}\ s^{-1}}{\overset{k_{on}^{PHH}=0.53\times10^{9}\ M^{-1}\ s^{-1}}{\rightleftharpoons}} PHH \qquad K_D=12\mu M$$

Supplementary Figure 2. Dependence of the exchange rates of ProTα (k_{ex}^{P}) and H1 (k_{ex}^{H}) on protein concentrations. (All dependencies calculated using the rate coefficients given in Supplementary Table 1.) Purple dotted lines show the exchange rates calculated using a two-state model for the formation of PH (Eq.17, see Methods for details). Solid purple lines show the exchange rates calculated using a kinetic model including the formation of the ternary complexes PPH and PHH (four-state model, Eqs. 13 and 16, see Methods for details). To identify whether the system is in slow or fast exchange under the experimental conditions, the exchange rates need to be compared with the observation timescales of the respective measurements, indicated as horizontal dashed lines for single-molecule FRET and NMR, respectively. The bottom panels show the calculated fraction of ProTα bound (in any of the complexes, PH, PPH, or PHH), θ_{P}^{bound}. The gray shading indicates protein concentrations outside the ranges at which the measurements were performed, or where one of the populations is present to <5 %, which would complicate the detection of line broadening, as indicated by the horizontal bars above the panels and in the lowest panels, respectively. **(a)** Conditions relevant for Figures 1b and 3b,c (single-molecule experiments). No deviation between two-state and four-state models is expected under these conditions since the populations of PPH and PHH are negligible, and slow-exchange behavior is expected in the experimentally accessible range. **(b, c, d)** Exchange rates for ProTα and H1 in the presence of 10 nM H1 (Figures 1c, 2a, and 3e,f, single-molecule experiments), 1 µM H1 (Figure 2b and Figure 4a,c, single-molecule and recurrence experiments), or 20 µM H1 (Figure 2c, single-molecule experiments), respectively, and varying concentrations of unlabeled ProTα (conditions of the corresponding experiments in Figures 1-3 shown above the panels). While the two-state model results in slow exchange between bound and unbound ProTα in all cases, the four-state model yields a transition from slow to fast exchange with increasing protein concentrations, in accord with the experimental data (Figure 2a,b,c). **(e)** Exchange rates of ProTα and H1 calculated for the experimental conditions used in the NMR titration of ^{15}N-ProTα with unlabeled H1 (Figure 2d-f). The two-state model results in slow exchange between bound and unbound ProTα on the NMR timescale, while the four-state model indicates fast exchange, in agreement with the experimental data (Figure 2e,f)

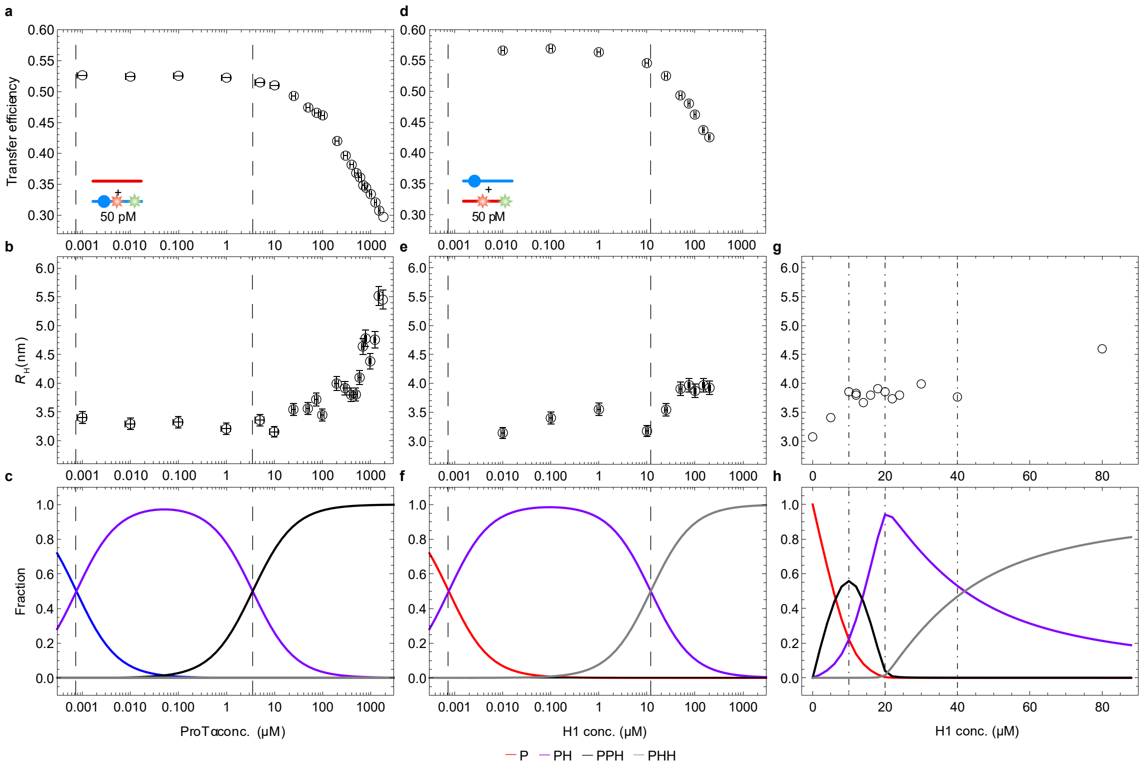

Supplementary Figure 3. Transfer efficiencies, hydrodynamic radii and peak intensity ratios indicate the formation of higher-order complexes. (a) Single-molecule FRET efficiencies of 50 pM donor/acceptor-labeled H1 in the presence of increasing concentrations of unlabeled ProTα. **(b)** Hydrodynamic radius (R_H) under the same conditions as in (a) from FCS. **(c)** Relative populations of P, PH, PPH and PHH, as a function of ProTα concentration under the conditions of (a, b) calculated according to the four-state model (Eq. 12, see Methods for details). Transfer efficiencies **(d)** and R_H from FCS **(e)** of 50 pM labeled ProTα in the presence of increasing concentration of unlabeled H1. Investigation of the H1 concentration range above 200 µM was prevented by high fluorescence background. **(f)** Relative populations of P, PH, PPH and PHH, as a function of H1 concentration under the conditions of (d, e) calculated according to the four-state model (Eq. 12, see Methods for details). Dashed vertical lines in A-F, from left to right, represent the K_D values for PH, PPH and PHH formation, respectively. **(g)** R_H from pulsed-field gradient NMR experiments with 20 µM ^{15}N-ProTα and increasing concentrations of unlabeled H1. **(h)** Relative populations of P, PH, PPH, and PHH as a function of the H1 concentration under the conditions of (g) calculated according to the four-state model (Eq. 12, see Methods for details, a-h: TBS, 200 mM ionic strength). Dash-dotted vertical lines represent the concentrations of H1 where the molar ratios H1 to ProTα are 1:2, 1:1, and 2:1, respectively (from left to right). In (a,b,d,e), horizontal error bars represent the estimated dilution error; in (b,e), vertical error bars are the result of propagating the standard error of the transfer efficiency.

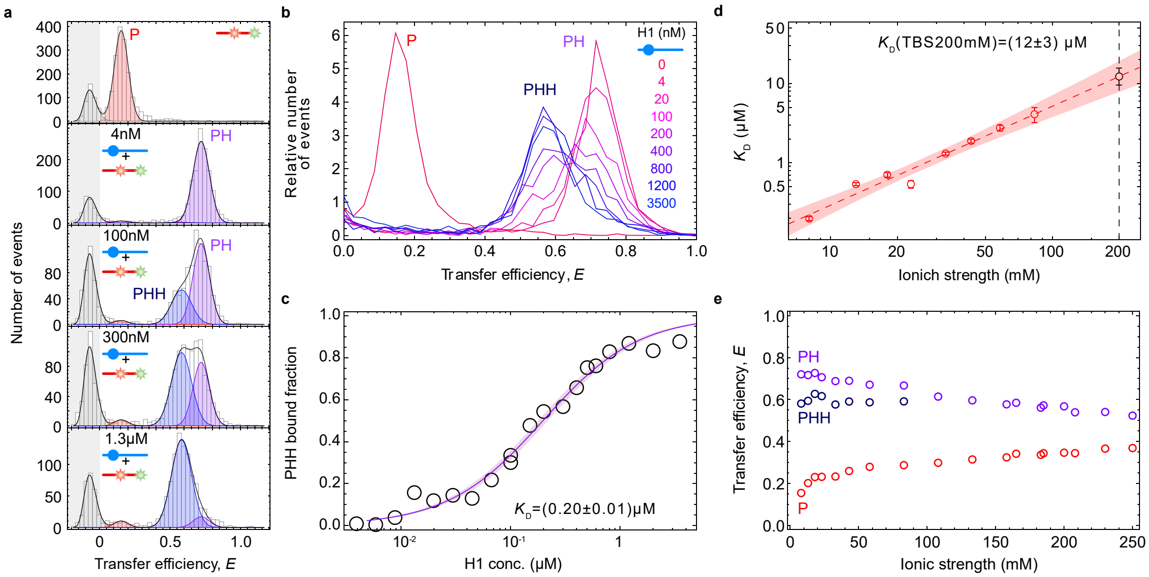

Supplementary Figure 4. Affinity and ionic-strength dependence of the ternary complex PHH. (a) Example of transfer efficiency histograms of 50 pM labeled ProTα with increasing concentrations of unlabeled H1 (concentrations indicated in the panels) in TBS at 8 mM ionic strength. The histograms show the peaks of unbound (P, red) and bound ProTα (PH purple, PHH blue), while the gray peak represents the donor-only population. **(b)** Superposition of the transfer efficiency histograms normalized by the number of events per measurement. The subpopulations P, PH and PHH are in slow exchange at this low ionic strength, as shown by the presence of separate peaks and an isosbestic point between the PH and PHH populations. **(c)** K_D for the reaction PH + H ⇌ PHH quantified by fitting the fraction of PHH ($c_{PHH}/(c_{PHH} + c_{PH})$ as a function of H1 concentration with a binding isotherm (Eq. 6, solid line). **(d)** The K_D of PHH was measured at different ionic strengths and extrapolated (dashed line with 90% confidence band), yielding a K_D of (12 ± 3) μM at 200 mM ionic strength. Vertical error bars show the standard errors of the fits of the PHH bound fraction as a function of the H1 concentration (see for example (c)). **(e)** Transfer efficiency of labeled ProTα in the unbound state (P, red), in PH (purple) and in the ternary complex PHH (dark blue) as a function of ionic strength.

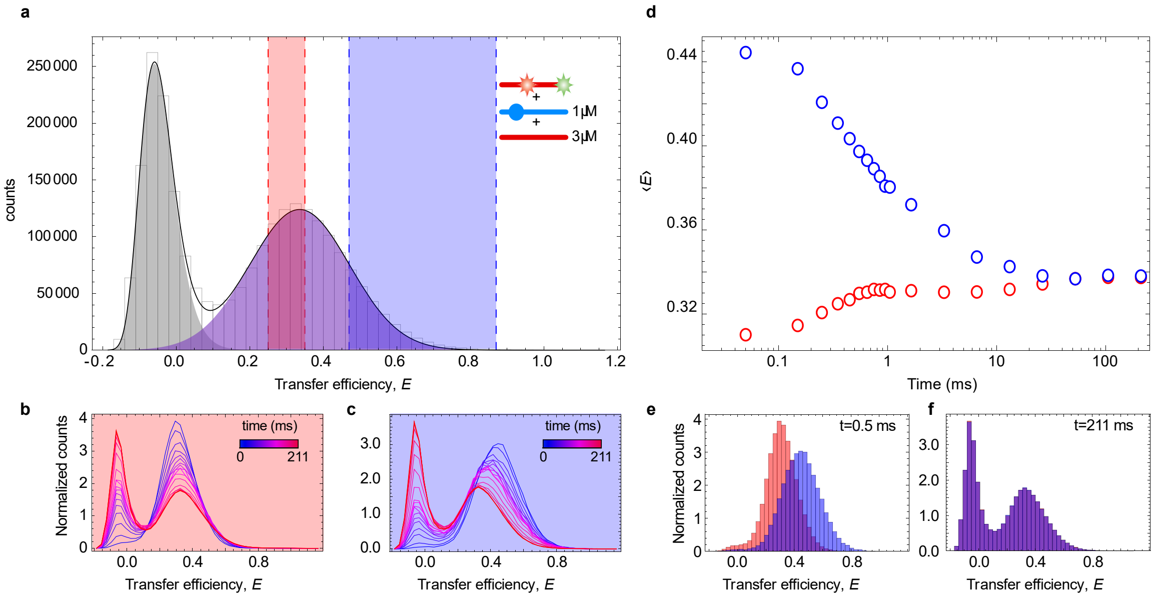

Supplementary Figure 5. Recurrence analysis of transfer efficiency histograms enables kinetic analysis in the fast exchange regime. (a) Transfer efficiency histogram of 150 pM double-labeled ProTα in the presence of 1 µM unlabeled H1 and 3 µM unlabeled ProTα. Two species are visible, the donor-only population (gray) and a FRET population (purple), comprising the time-averaged signal from unbound (P) and bound (PH, PPH and PHH) labeled ProTα. The red- and blue-shaded areas represent the transfer efficiency intervals used to select the initial bursts for recurrence analysis. **(b, c)** Transfer efficiency histograms generated with increasing delay time (see Methods for details) from bursts selected in the corresponding transfer efficiency intervals shown above. With increasing delay time, the donor-only population increases due to photobleaching of the acceptor, and the position of the FRET population shifts, relaxing towards the equilibrium transfer efficiency histogram. **(d)** The transfer efficiency of the FRET population as a function of delay time, showing that the system equilibrates in less than 100 ms. **(e, f)** Examples of transfer efficiency histograms after delay times of 50 µs and 211 µs, respectively, show the separation in transfer efficiencies of the initial populations for short recurrence times and their full convergence for long times.

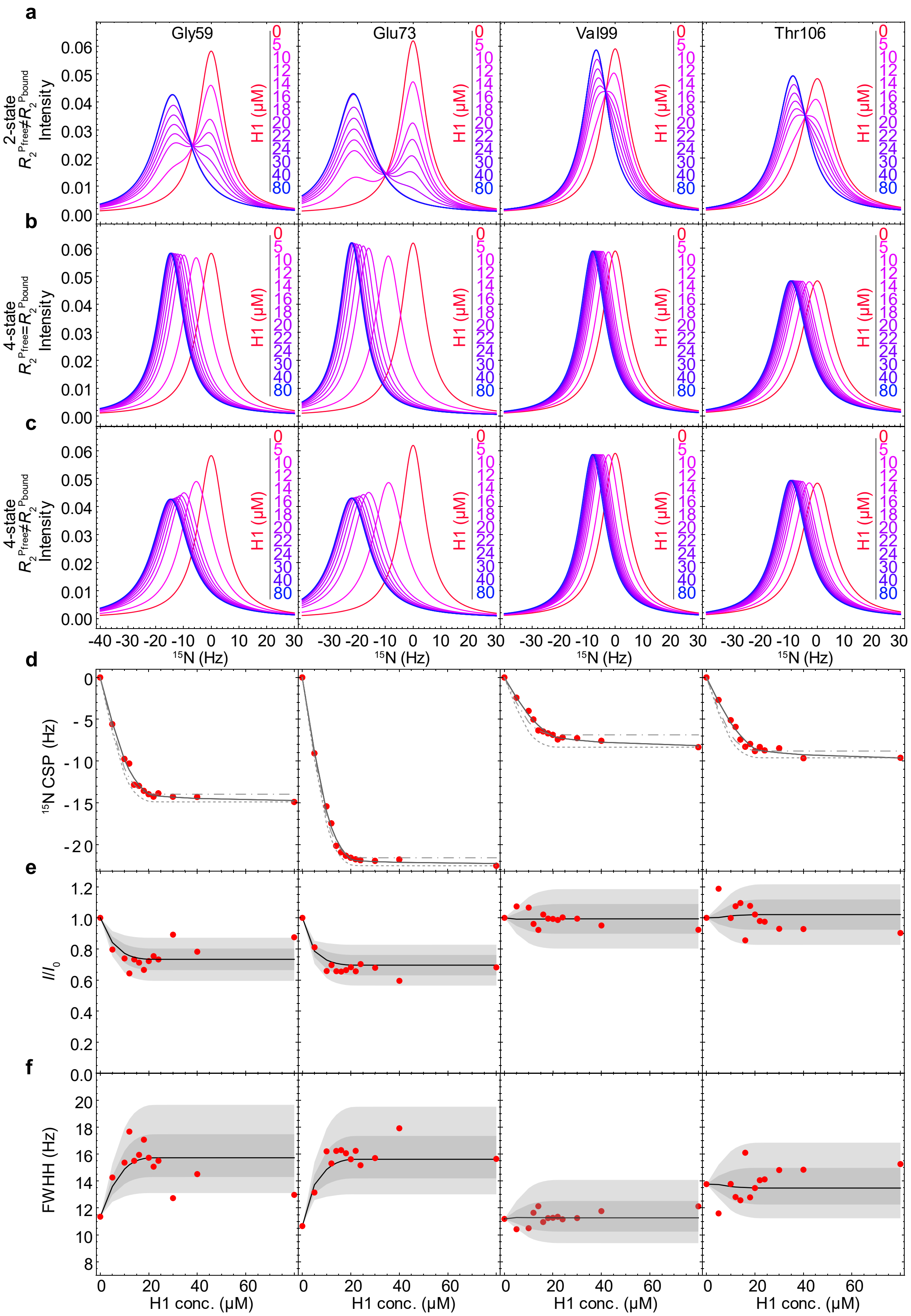

a
b
c
d
e
f
Gly59
Glu73
Val99
Thr106
H1 (μM)
0
5
10
12
14
16
18
20
22
24
30
40
80
2-state
4-state
Intensity
$R_2^{P_{free}} \neq R_2^{P_{bound}}$
$R_2^{P_{free}} = R_2^{P_{bound}}$
^{15}N (Hz)
^{15}N CSP (Hz)
I/I_0
FWHH (Hz)
H1 conc. (μM)

Supplementary Figure 6. NMR lineshape calculations. The lineshapes of individual NMR residues (indicated in the panels) were calculated using the Bloch-McConnell equation for different kinetic models (see Methods for details). **(a)** Lineshapes calculated assuming a two-state model (Eq. 9). Two peaks in slow exchange are visible. **(b)** Lineshapes calculated for the four-state model including the ternary complexes, and assuming the same linewidth for the sup-populations of P, PH, PPH, and PHH (linewidth obtained from the spectra of ProTα in the absence of H1). This analysis shows a single peak shifting with increasing concentration of H1, in agreement with the fast exchange observed in the NMR experiments, but in disagreement with the broadening observed during the H1 titration (Figure 2f). **(c)** To account for the line broadening with increasing concentration of H1, we set the linewidth of the bound ProTα subpopulations PH, PPH, PHH to the value from the NMR spectra at 20 μM H1 (1:1 molar ratio of ProTα:H1), where the fraction of PH is expected to be close to 100% (FigureSupplementary Figure 3h). The resulting analysis yields good agreement with the NMR experiments (Figure 2). **(d,f)** Comparison of peak positions, intensity ratios (I/I_0), and full width at half height (FWHH) from the Bloch-McConnell calculation, using the approach described in (c), with the experimental data (red points). **(d)** Calculated chemical shifts as a function of H1 concentration show good agreement with the experimental data (red symbols), independent of whether we choose individual Larmor frequencies ω for each sub-population P, PH, PPH, PHH (full black line) (see Methods for details) or whether the Larmor frequency of the ProTα-bound subpopulations (PH, PPH, PHH) were assumed to be equal ($\omega_{PH} = \omega_{PPH} = \omega_{PHH} = \omega_{Pbound}$) (dot-dashed gray line for $\omega_{Pbound} = \omega(c_H{=}20)$ and dashed gray line for $\omega_{Pbound} = \omega(c_H{=}80)$). **(e,f)** I/I_0 (e) and FWHH (f) from calculated lineshapes. Shaded gray areas show error bands assuming 10% (dark gray) or 20% (light gray) uncertainty in the line widths of the bound ProTα subpopulations.

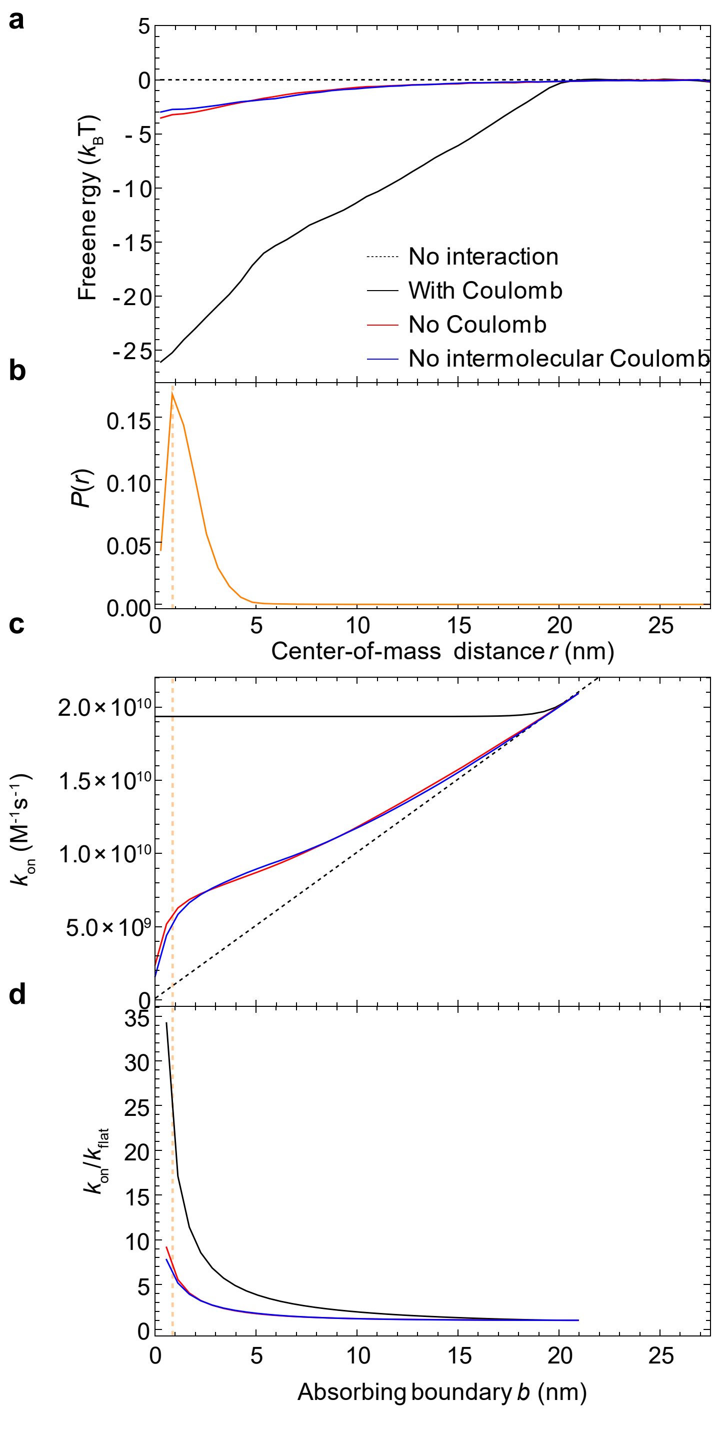

Supplementary Figure 7. Association rate coefficients from potentials of mean force (PMFs). (a) PMFs for association of ProTα and H1 from coarse-grained umbrella sampling simulations with and without Coulomb interactions (see legend) as a function of the center-of-mass distance, *r*, between the proteins. **(b)** Radial probability density, *P*(*r*), with the vertical orange dashed line indicating the maximum. **(c)** Association rate coefficients, k_{on}, calculated from PMFs in (a) and the experimentally determined translational diffusion coefficients of H1 and ProTα to an absorbing boundary, *b*, at a given intermolecular separation (see Methods for details). **(d)** Ratio of k_{on} for different scenarios over the association rate coefficient without intermolecular interactions, k_{flat}.

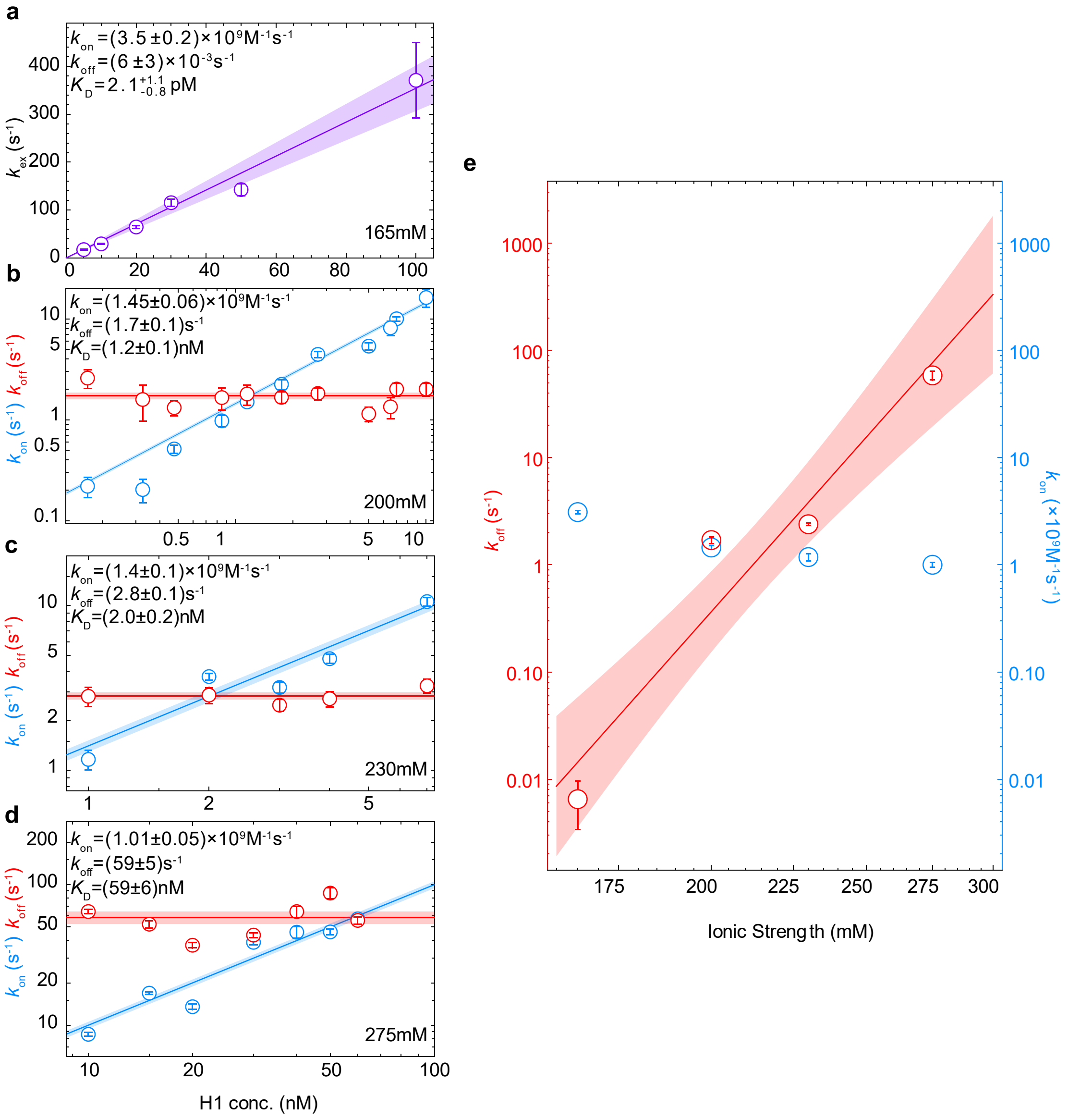

Supplementary Figure 8. Ionic-strength dependence of interaction kinetics under two-state conditions. (a) Relaxation rates from stopped-flow experiments in TBS 165 mM ionic strength with 2 nM labeled ProTα mixed with different concentrations of H1. The observed rate, k_{ex}, increases linearly as a function of H1 concentration. Purple line and shaded area represent the fit with $k_{ex} = k_{on}c_H + K_D k_{on}$ and 95% confidence interval, respectively. Error bars are the standard errors of the fits of the averaged signal of at least 80 repeats. **(b-d)** Results from single-molecule surface-immobilization experiments in TBS at ionic strengths from 200 mM to 275 mM analogous to Figure 3a, analyzed with a two-state binding model. The error bars are the s.d. estimated from bootstrapping analysis (see Materials and Methods for more details). **(e)** The resulting association and dissociation rate coefficients as a function of ionic strength. While the association rate coefficient is only weakly dependent on ionic strength, the change in dissociation rate coefficient dominates the change in K_D. A fit of k_{off} with the Record/Lohman model[2] suggests the binding of 17 ± 3 counterions upon dissociation, close to the value from a corresponding analysis of the K_D (Supplementary Figure 1). Error bars represent standard errors of the fits at each ionic strength.[1]

K_D (nM)

		Ionic Strength (mM)									
	Variants	165	180	200	208	230	240	275	290	330	340
$P + H \rightleftharpoons PH$	ProTα E56C/D110C Alexa488/594	$(2.1^{+1.1}_{-0.8}) \times 10^{-3}$	$(3.7 \pm 0.5) \times 10^{-2}$	0.73 ± 0.07	1.0 ± 0.1		25 ± 3		$(2.3 \pm 1.5) \times 10^{2}$	$(1.4 \pm 0.4) \times 10^{3}$	$(4 \pm 2) \times 10^{3}$
	ProTα S1C/D110C Alexa488/594				2.0 ± 0.1						
	ProTα E56C/D110C Cy3B/Abb.STAR635				1.0 ± 0.1						
	ProTα E56C/D110C Atto 550/647N				3.1 ± 0.2						
	H1 V104C/G194C Alexa488/594				3.5 ± 0.2						
	ProTα S1C/D109C Alexa488/594				2.3 ± 0.5						
	ProTα S1C/E55C Alexa488/594				1.3 ± 0.2						
	ProTα E55C/D109C Alexa488/594			0.51 ± 0.05	1.3 ± 0.1						
	Avi_ProTα E56C/D110C Cy3B-LD650 free diffusion			1.12 ± 0.08							
	Avi_ProTα E56C/D110C Cy3B-LD650 surface			1.2 ± 0.2		2.0 ± 0.2		59 ± 6			
	Unlabeled ProTα	$(5.8 \pm 0.6) \times 10^{-3}$		1.1 ± 0.4		6.5 ± 0.4					
$PH + P \rightleftharpoons PPH$	ProTα E56C/D110C Alexa488/594			$(3.5 \pm 0.4) \times 10^{3}$							

		Ionic Strength (mM)								
	Variants	8	13	18	23	33	43	58	83	200
$PH + H \rightleftharpoons PHH$	ProTα E56C/D110C Alexa488/594	$(2.0 \pm 0.1) \times 10^{2}$	$(5.4 \pm 0.2) \times 10^{2}$	$(7.1 \pm 0.4) \times 10^{2}$	$(5.4 \pm 0.6) \times 10^{2}$	$(1.31 \pm 0.06) \times 10^{3}$	$(1.8 \pm 0.1) \times 10^{3}$	$(2.8 \pm 0.2) \times 10^{3}$	$(4.1 \pm 0.9) \times 10^{3}$	$(12 \pm 3) \times 10^{3}$

Supplementary Table 1. Comparison of equilibrium dissociation constants, K_D, of ProTα and H1 for different labeling variants and dye pairs at different ionic strengths[1] and for the different complexes (see equilibria on the left).

			Ionic Strength (mM)			
			**165**	**200**	**230**	**275**
Rates	$P + H \rightleftharpoons PH$	$k_{on}\ (M^{-1}s^{-1})$	$(3.5 \pm 0.2) \times 10^9$	$(1.45 \pm 0.06) \times 10^9$	$(3.1 \pm 0.1) \times 10^9$	$(3.1 \pm 0.1) \times 10^9$
		$k_{off}(s^{-1})$	$(6 \pm 3) \times 10^{-3}$	(1.7 ± 0.1)	(2.8 ± 0.1)	(59 ± 5)
	$PH + P \rightleftharpoons PPH$	$k_{on}^{PPH}\ (M^{-1}s^{-1})$		$(0.53 \pm 0.1) \times 10^9$		
		$k_{off}^{PPH}\ (s^{-1})$		$(1.9 \pm 0.2) \times 10^3$		
	$PH + H \rightleftharpoons PHH$	$k_{on}^{PHH}\ (M^{-1}s^{-1})$		$(0.53 \pm 0.1) \times 10^9$		
		$k_{off}^{PHH}\ (s^{-1})$		$(6 \pm 2) \times 10^3$		

Supplementary Table 2. Comparison of association and dissociation rate coefficients of ProTα and H1 at different ionic strengths and for the different complexes (see equilibria on the left).

ProTα (unlabeled & NMR)	1<br>MAHHHHHHSAALEVLFQ/GPM**S**DAAVDTSSEITTKDLKEKKEVVEEAENGRDAPANGNANEENGEQEADNEVDEEEEEGGEEEEEEEEGDGEEEDGDEDE<br>EAESATGKRAAEDDEDDDVDTKKQKTDEDD
ProTα S1C/E55C	1<br>MAHHHHHHSAALEVLFQ/GPM**C**DAAVDTSSEITTKDLKEKKEVVEEAENGRDAPANGNANEENGEQEADNEVDEE**C**EEGGEEEEEEEEGDGEEEDGDEDE<br>EAESATGKRAAEDDEDDDVDTKKQKTDEDD
ProTα S1C/D109C	1<br>MAHHHHHHSAALEVLFQ/GPM**C**DAAVDTSSEITTKDLKEKKEVVEEAENGRDAPANGNANEENGEQEADNEVDEEEEEGGEEEEEEEEGDGEEEDGDED<br>EEAESATGKRAAEDDEDDDVDTKKQKTDED**C**
ProTα E55C/D109C	1<br>MAHHHHHHSAALEVLFQ/GPM**S**DAAVDTSSEITTKDLKEKKEVVEEAENGRDAPANGNANEENGEQEADNEVDEE**C**EEGGEEEEEEEEGDGEEEDGDEDE<br>EAESATGKRAAEDDEDDDVDTKKQKTDED**C**
ProTα E56C/D110C	1<br>MAHHHHHHSAALEVLFQ/GP**S**DAAVDTSSEITTKDLKEKKEVVEEAENGRDAPANGNAENEENGEQEADNEVDEE**C**EEGGEEEEEEEEGDGEEEDGDEDE<br>EAESATGKRAAEDDEDDDVDTKKQKTDED**C**
ProTα S1C/D110C	1<br>MAHHHHHHSAALEVLFQ/GP**C**DAAVDTSSEITTKDLKEKKEVVEEAENGRDAPANGNAENEENGEQEADNEVDEEEEEGGEEEEEEEEGDGEEEDGDEDE<br>EAESATGKRAAEDDEDDDVDTKKQKTDED**C**
Avi-ProTα E56C/D110C	1<br>*MAGLNDIFEAQKIEWHEGSMGSGS*M**S**DAAVDTSSEITTKDLKEKKEVVEEAENGRDAPANGNAENEENGEQEADNEVDEE**C**EEGGEEEEEEEEGDGEEED<br>GDEDEEAESATGKRAAEDDEDDDVDTKKQKTDED**C**GGPR/GSRSQASHHHHHH
Unlabeled H1	1<br>**T**ENSTSAPAAKPKRAKASKKSTDHPKYSDMIVAAIQAEKNRAGSSRQSIQKYIKSHYKVGENADSQIKLSIKRLVTTGVLKQTKGVGASGSFRLAKSDEPKKSV<br>AFKKTKKEIKKVATPKKASKPKKAASKAPTKKPKATPVKKAKKKLAATPKKAKKPKTVKAKPVKASKPKKAKPVKPKAKSSAKRAGKKK
H1-V104C-G194C	1<br>M**T**ENSTSAPAAKPKRAKASKKSTDHPKYSDMIVAAIQAEKNRAGSSRQSIQKYIKSHYKVGENADSQIKLSIKRLVTTGVLKQTKGVGASGSFRLAKSDEPKK<br>S**C**AFKKTKKEIKKVATPKKASKPKKAASKAPTKKPKATPVKKAKKKLAATPKKAKKPKTVKAKPVKASKPKKAKPVKPKAKSSAKRAGKKK**C**GPR/GSRSQ<br>ASHHHHHH

Supplementary Table 3. Amino acid sequences of proteins used. Protease cleavage sites for His_6 tags are indicated by a slash, the avi-tag with flexible linker in italics, Cys residues introduced for labeling and amino acid number 1 are indicated in bold for each sequence. ProTα (unlabeled & NMR), ProTα S1C/E55C, ProTα S1C/D109C, ProTα E55C/D109C are variants of human ProTα isoform 2, while ProTα E56C/D110C, ProTα S1C/D110C, and Avi-ProTα E56C/D110C are variants of isoform 1. The isoforms differ by a single Glu at position 39.

Supplementary References

1 Borgia, A. *et al.* Extreme disorder in an ultrahigh-affinity protein complex. *Nature* **555**, 61-66, doi:10.1038/nature25762 (2018).

2 Record, M. T., Jr., Anderson, C. F. & Lohman, T. M. Thermodynamic analysis of ion effects on the binding and conformational equilibria of proteins and nucleic acids: the roles of ion association or release, screening, and ion effects on water activity. *Q. Rev. Biophys.* **11**, 103-178, doi:DOI: 10.1017/s003358350000202x (1978).
